# Uncommon *N*-Glycan Structures in Anhydrobiotic Tardigrades

**DOI:** 10.1016/j.mcpro.2025.100979

**Published:** 2025-04-28

**Authors:** Hirokazu Yagi, Taiki Saito, Shih-Yun Guu, Nao Yamakawa, Shigeru Shimamura, Sachiko Kondo, Maho Yagi-Utsumi, Ken Takai, Jun-ichi Furukawa, Yann Guérardel, Kay-Hooi Khoo, Kazuharu Arakawa, Koichi Kato

**Affiliations:** 1 Graduate School of Pharmaceutical Sciences, Nagoya City University, Nagoya, Japan; 2 Department of Creative Research, Exploratory Research Center on Life and Living Systems (ExCELLS), National Institutes of Natural Sciences, Okazaki, Japan; 3 Institute of Biological Chemistry, Academia Sinica, Taipei, Taiwan; 4 Université de Lille, CNRS, INSERM, CHU Lille, Institut Pasteur de Lille, US 41-UAR 2014-PLBS, Lille, France; 5 Université de Lille, CNRS, UMR 8576 - UGSF-Unité de Glycobiologie Structurale et Fonctionnelle, Lille, France; 6 Institute for Glyco-Core Research, Nagoya University, Nagoya, Japan; 7 Institute for Extra-cutting-edge Science and Technology Avant-garde Research (X-star), Japan Agency for Marine-Earth Science and Technology (JAMSTEC), Yokosuka, Kanagawa, Japan; 8 Institute for Glyco-Core Research, Gifu University, Gifu, Japan; 9 Institute for Advanced Biosciences, Keio University, Tsuruoka, Yamagata, Japan; 10 Graduate School of Media and Governance and Faculty of Environment and Information Studies, Keio University, Fujisawa, Kanagawa, Japan; 11 Institute for Molecular Science, National Institutes of Natural Sciences, Okazaki, Japan

**Keywords:** fucosylation, N-Glycan, glycomics, glycoproteomics, tardigrade

## Abstract

We characterized the *N*-glycosylation profiles of anhydrobiotic tardigrades, *Ramazzottius varieornatus* and *Hypsibius exemplaris*, identifying high-mannose, paucimannose, and complex-type oligosaccharides, while hybrid-type glycans were undetectable. Notably, paucimannose-type oligosaccharides accounted for 39% of the *N*-glycans in *R. varieornatus* and 17% in *H. exemplaris*, with a substantial proportion of them exhibiting fucosylation of the innermost GlcNAc via an α1,6-linkage. This core fucosylation pattern, common to all animals, was observed alongside a distinctive glycosylation signature prominently observed in tardigrades: complex-type glycans lacking galactosylation but containing α1,3-fucosylated GlcNAc at non-reducing termini. This structure was more prevalent in *H. exemplaris*, with 22 out of 87 identified glycoproteins expressing the Fucα1,3-GlcNAc motif, including eight induced during anhydrobiosis. Key glycoproteins such as Cu/Zn-superoxide dismutase and papilin, implicated in oxidative stress protection and extracellular matrix remodeling, were among those modified. Comparative analyses reveal that non-reducing terminal α1,3-fucosylation in tardigrades is distinct from the mammalian Lewis X antigen and similar structures found in invertebrates, suggesting a unique substrate specificity of fucosyltransferases in these species. Genomic analysis identified homologs of FUT9 and FucTC, indicating potential candidates responsible for this glycosylation pattern. Our findings provide new insights into the molecular mechanisms of glycosylation in tardigrades and their relevance to their extreme stress tolerance.

## Highlights

• Paucimannose-type glycans dominate *N*-glycosylation in tardigrades.
• Uncommon antennal Fucα1,3-GlcNAc structure is prevalent in *Hypsibius exemplaris.*
• Fucα1,3-GlcNAc motif is found in certain glycoproteins induced during anhydrobiosis.

## Graphical Abstract

**Figure:**
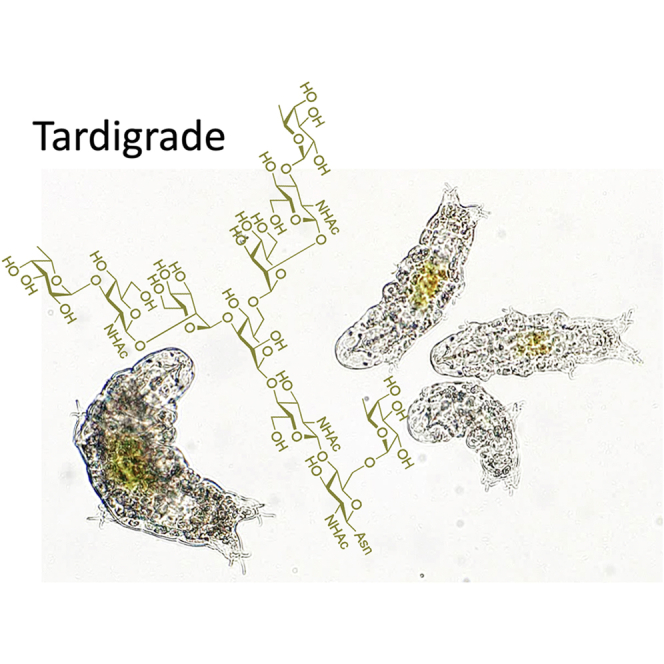

## In Brief

This study identifies paucimannose-type glycans as dominant *N*-glycosylation and uncommon N-glycans with Fucα1,3-GlcNAc motifs in anhydrobiotic tardigrades. These Fucα1,3-GlcNAc structures are associated with proteins involved in oxidative stress protection and extracellular matrix remodeling. Genomic analyses suggest specific fucosyltransferases responsible for this glycosylation. These findings offer insights into the molecular mechanisms underlying tardigrade resilience and highlight the potential role of glycans in surviving extreme stress.

Tardigrades are members of the superphylum Ecdysozoa, which includes molting invertebrates (1). Over 1400 species of tardigrades have been discovered in various environments such as the sea, land, and lakesides, thriving under wet conditions (2). Many terrestrial tardigrades can adapt to dry conditions through anhydrobiosis, a state where metabolic activity ceases due to extreme water deprivation. In this anhydrobiotic state, tardigrades exhibit remarkable tolerance to various extreme environmental conditions, including extreme temperatures, radiation, vacuum, and toxic chemicals (3, 4, 5, 6, 7). This process is reversed, and they immediately return to their normal metabolic state upon rehydration.

In recent years, significant progress has been made in understanding the molecular mechanisms underlying tardigrade stress tolerance. The establishment of standard culture systems for species such as *Ramazzottius varieornatus* and *Hypsibius exemplaris* has facilitated genetic and biochemical analyses (8, 9). Several proteins have been identified that are believed to play key roles in anhydrobiosis and stress tolerance, including cytoplasmic abundant heat soluble (CAHS) proteins, secretory abundant heat soluble (SAHS) proteins, mitochondrial late embryogenesis abundant (LEAM) proteins, and mitochondrial abundant heat soluble (MAHS) proteins (7, 10, 11, 12, 13). However, the precise mechanisms governing anhydrobiosis in tardigrades remain to be fully elucidated.

*N*-Glycosylation is a major post-translational modification that regulates cell-cell communication among various cell types. *N*-glycans in eukaryotes share a common core structure consisting of three mannose (Man) and two *N*-acetylglucosamine (GlcNAc) residues, with species-specific structures at the non-reducing ends. *N*-glycan structures vary in size, components, and branching patterns, reflecting the organism’s physiological response to its environment. In tardigrades, these *N*-glycans are likely to contribute to their extraordinary survivability in extreme environments by mediating various cellular processes.

In this study, we report the detailed *N*-glycan profiles of two closely related tardigrade species, *H. exemplaris* and *R. varieornatus* (both members of the superfamily Hypsibioidea). Our analysis reveals the presence of distinctive glycan structures in these tardigrades. Additionally, we identified specific glycoproteins bearing these carbohydrate moieties, some of which are known to be induced under desiccation conditions.

## Experimental Procedures

### Tardigrade Culture

The tardigrades, *H. exemplaris* and *R. varieornatus*, were cultured as previously described (8, 14). In summary, they were nourished with *Chlorella vulgaris* (Chlorella Industry) on 2% Bacto Agar (Difco) plates and maintained at 18 °C for *H. exemplaris* and 22 °C for *R. varieornatus* under dark conditions. Anhydrobiotic adult specimens were placed within a controlled chamber with relative humidity gradients. The criterion for successful anhydrobiosis was set at >90% recovery rate after rehydration, as observed across samples prepared within the same chamber.

### Preparation of Fluorescence-Tagged *N*-glycans

All experimental procedures for profiling, including chromatographic conditions and matrix-assisted laser desorption/ionization time-of-flight mass spectrometry (MALDI-TOF-MS) and MS/MS techniques, have been previously described (15, 16, 17).

*N*-glycans were released from glycoproteins obtained from 6000 tardigrades in an anhydrobiotic state through liquid-phase hydrazinolysis, followed by pyridylamination. Initially, the dried material was sequentially extracted three times using chloroform/methanol mixtures in ratios of 2:1 (v/v) and 1:2 (v/v). The pellet fraction, which contained the glycoproteins, was then resuspended in 20 mM Tris-HCl buffer (pH 8.0) and centrifuged at low speed to remove insoluble debris. The resulting supernatant was precipitated by adding 70% ethanol at 4 °C and incubating overnight. The precipitate was collected by centrifugation at 1200 *g* as a protein pellet.

This protein pellet was dried using a rotary vacuum evaporator and subsequently incubated with 200 μl of hydrazine anhydride in a screw-capped glass tube at 95 °C for 10 h to release the glycans. The reaction was quenched by mixing with 3 ml of 50 mM ammonium acetate buffer (pH 7.0), and then the mixture was loaded onto a graphite carbon column (GL-Pak Carbograph 150 mg/3 ml, GL Sciences) that had been pre-equilibrated with the same buffer. After washing the column with 5 ml of ammonium acetate buffer and 5 ml of triethylamine acetate buffer (40% acetonitrile, pH 7.0), the glycans were eluted using 5 ml of triethylamine acetate buffer containing 2% acetic anhydride. The eluate was dried using a rotary vacuum evaporator to yield *N*-acetylated glycans.

For pyridylamination, a labeling solution was prepared by dissolving 276 mg of 2-aminopyridine in 100 μl of acetic acid. The dried glycan sample was dissolved in 30 μl of the labeling solution and incubated at 90 °C for 60 min in a tightly sealed screw-capped glass tube. Subsequently, a reducing solution, consisting of 200 mg of dimethylamine-borane dissolved in a mixture of 80 μl acetic acid and 50 μl ultrapure water, was added to the reaction tube. The mixture was incubated at 80 °C for 35 min to complete the reaction.

To purify the pyridylaminated (PA) glycans, the reaction mixture was applied to a cellulose column equilibrated with buffer I (66% 1-butanol, 16% ethanol, 16% 0.6 M acetic acid). The column was washed twice with the same buffer before glycans were eluted with 2 ml of buffer II (33% ethanol, 66% 75 mM ammonium bicarbonate). The eluate was then dried using a SpeedVac vacuum concentrator, yielding purified PA-labeled glycans suitable for further analysis. The resulting PA-glycans were then dried, redissolved in double-distilled water, and subjected to HPLC analysis.

### Fractionation and Structural Analysis of PA-Glycans by HPLC

The PA-glycan sample mixture was fractionated using reverse-phase HPLC with a Shim-pack HRC octadecyl silica (ODS) column (6.0 mm inner diameter x 150 mm; Shimadzu, Kyoto, Japan). PA-labeled glycans were detected by fluorescence at excitation and emission wavelengths of 320 nm and 400 nm, respectively. Elution was performed at a flow rate of 1.0 ml/min at 55 °C using two solvents: A and B. Solvent A was 10 mM sodium phosphate buffer (pH 3.8), and solvent B was 10 mM sodium phosphate buffer (pH 3.8) containing 0.5% 1-butanol. The column was equilibrated with 80% solvent A and 20% solvent B. The gradient elution was set from 0 to 60 min with a linear gradient of 20 to 50% solvent B. PA-labeled glucose oligomers (Takara Bio Inc., Shiga, Japan), composed of glucose units linked via α-1,6-glycosidic bonds with defined degrees of polymerization (DP4–DP21), were analyzed in advance using ODS columns. The elution times were converted to glucose units (GU), which reflect the degree of polymerization. This conversion allows for normalization of retention times, compensating for lot-to-lot variation and column degradation, thereby ensuring analytical consistency.

Each PA-glycan fraction was separated based on differences in ODS elution time, attributed to the intrinsic chemical structure of the glycans. Each fractionated PA-glycan sample was mixed with a matrix solution (10 mg/ml dihydrobenzoic acid) and analyzed using MALDI-TOF-MS on an Autoflex Speed instrument (Bruker Daltonics) in positive-ion reflector mode. For MS acquisition, the PA glycans in water were mixed with dihydrobenzoic acid (10 mg/ml in 50% acetonitrile) at a 1:1 ratio, and the resulting mixture was spotted onto the target plate. The instrument settings included a laser intensity of 50 to 78%, resulting in a total of 5000 shots for linear positive mode MS. The MS survey and data acquisition were performed manually. By comparing the mass values of PA-glycans and their elution positions on the ODS column with data in the GALAXY database (18, 19), the structure and/or components of each *N*-glycan fraction were anticipated. This method enables the identification of glycan isomers, making the resulting data essential for interpreting the glycoproteomics data obtained from the nanoLC-MS/MS analysis of glycopeptides described below. PA-glycans that did not match any of the previously registered *N*-glycans in the GALAXY database were trimmed using α-fucosidase from bovine kidney (Sigma), α-mannosidase (Seikagaku Co.), and/or β-*N*-acetylglucosaminidase S, which was cloned from *Streptococcus pneumoniae* and expressed in *E. coli*. (New England Biolabs), to align their structures with known forms. To identify the PA-glycans in peaks 8 and 11, MS/MS analysis was conducted in positive ion mode using a Bruker UltrafleXtreme MALDI-TOF/TOF mass spectrometer. The analysis employed LIFT mode under reflector positive ion conditions. The acquired mass spectra were processed using flexAnalysis 3.4 software (Bruker Daltonics).

### MALDI-TOF-MS and MS/MS Analysis of Permethylated PA-glycans

PA-glycans with mass values indicating the presence of fucose and *N*-acetylglucosamine residues at the non-reducing end, but lacking galactose residues, were further permethylated using the NaOH/dimethyl sulfoxide slurry method as previously described (15, 16). The permethylated sample aliquots were mixed 1:1 with matrix solution of 10 mg/ml 2,5-dihydroxybenzoic acid in 50% acetonitrile for positive mode analysis, and then spotted onto the MALDI plate for data acquisition using an AB SCIEX MALDI TOF/TOF 5800 system. The instrument settings included a laser intensity of 4500 with 50 subspectra, each consisting of 100 shots, resulting in a total of 5000 shots for reflector positive mode MS1. For MS/MS analysis in reflector positive mode at 2 kV CID energy, the laser intensity was set to 5000 under the same subspectral conditions. To enhance the signal-to-noise ratio, multiple MS/MS spectra of the same precursor were combined to generate the final spectrum. The mass resolution for the MS mode was approximately 16,000 for peaks around *m/z* 1500 and 5000 for MS2 fragment ions around m/z 1000. The precursor mass window was configured at 200 resolution (FWHM), with the metastable suppressor activated.

The MS survey and data acquisition were conducted manually. All permethylated glycans and glycan fragments were identified as [M+Na]^+^ sodium adducts. Specific MS ions were selected as parent ions for MS/MS fragmentation pattern acquisition after examining the summed MALDI-TOF-MS spectra (15, 16).

### Gas Chromatography-Electron Impact MS (GC-EI-MS) Methylation Analyses

Approximately 200 pmol of PA-labeled glycan mixtures from *H. exemplaris* and *R. varieornatus* were permethylated using the NaOH/dimethyl sulfoxide slurry method, as described in the previous study (20). The permethylated oligosaccharides were hydrolyzed with 4 M trifluoroacetic acid at 100 °C for 4 h, followed by reduction with 10 mg/ml sodium borodeuteride (NaBD_4_) for 16 h at room temperature. Subsequently, the resulting products were acetylated to yield the partially methylated alditol acetates (PMAA).

Gas chromatography (GC) was performed on a Trace GC Ultra system (Thermo Scientific) equipped with a SOLGEL-1MS capillary column (30 m × 0.25 mm ID, 0.25 μm film thickness) and detected by a TSQ Quantum GC detector (Thermo Scientific). The carrier gas was helium (99.9995%) at a constant pressure of 100 kPa and an oven temperature of 120 °C. The temperature gradient was set from 120 °C to 230 °C over 55 min at a rate of 2 °C/min, and then increased to 270 °C in 4 min at a rate of 10 °C/min. An electron ionization source mode at 70 eV was used for the mass spectrometer operation. The mass spectral data and retention times of the PMAA derivatives of 4-GlcNAc, 3-GlcNAc, and non-reducing terminal GlcNAc were compared with those of authentic standard compounds for identification.

### NanoLC-MS Measurements of Tardigrade Glycopeptides

For the identification of glycoproteins carrying a specific glycotope, Fucα1,3-GlcNAc, we conducted nanoLC-MS/MS analysis on protein fractions derived from *H. exemplaris*. Proteins were extracted from approximately 10,000 tardigrades, alkylated, and digested with trypsin. The extraction involved sequentially treating the dried material with its lipid was sequentially extracted from the cells with 80% ethanol, 100% ethanol, chloroform/methanol (2:1, v/v), chloroform/methanol/H_2_O (1:2:0.8, v/v/v), and 80% acetone, as previously described (21). The proteins were then dissolved in 500 μl of Milli-Q water, denatured with 0.1% Rapigest (Waters) in 50 mM Tris–HCl, reduced with DTT (Fujifilm Wako), alkylated with iodoacetamide (Fujifilm Wako), and digested with Lys-C (Fujifilm Wako) at a ratio of 1:200 (weight of protein) and sequencing-grade modified trypsin (Promega) at a ratio of 1:100 (weight of protein) at 37 °C overnight in 0.1% Rapigest. After digestion, Rapigest was removed by acidification with 0.1% trifluoroacetic acid (TFA) (Fujifilm Wako).

The resulting peptides were then loaded onto an amide column (TSK-gel Amide-80, 2 × 50 mm; TOSOH) to enrich glycopeptides, following the method outlined in a previous study (22). Glycopeptides from tryptic digests were captured using an Amide-80 column that had been equilibrated with 75% acetonitrile (Merck) and 0.1% TFA and were subsequently eluted isocratically with 50% acetonitrile and 0.1% TFA after washing.

The glycoprotein fraction was then analyzed using an Orbitrap Fusion Tribrid mass spectrometer (Thermo Fisher Scientific) coupled with an UltiMate 3000 RSLCnano Nanoflow liquid chromatography system (Thermo Fisher Scientific). Glycopeptide separations were performed on a reverse-phase column (Zaplous alpha Pep-C18, 3 μm, 120 Å, 0.1 × 150 mm, AMR Inc) using a linear gradient of 5%-45% solvent B over 220 min at a constant flow rate of 500 nl/min. Solvent A was 0.1% formic acid, and solvent B was 100% acetonitrile. A nano-electrospray ion source (Dream spray, AMR Inc.) was used, with the column oven set to 35 °C.

The Orbitrap Fusion Tribrid mass spectrometer operated in positive ion mode with an electrospray voltage of 1.6 kV and an ion transfer tube temperature of 250 °C. Full scan MS spectra were acquired in the Orbitrap mass analyzer (*m/z* range: 350–1800, resolution: 120,000 FWHM) using internal calibration (EASY-IC). MS/MS spectra were obtained in the Orbitrap mass analyzer (mass range: normal, scan range mode: auto, resolution: 50,000 FWHM) using higher-energy collisional dissociation (HCD) with a normalized collision energy of 28% for efficient detection of signature ions. The HCD fragment ions corresponding to glycan oxonium fragments (HexNAc: *m/z* 204.0867, HexNAc-Hex: *m/z* 366.1396, HexNAc-DHex: *m/z* 350.1395, HexNAc fragment: *m/z* 138.0545) were used to trigger EThcD/CID fragmentation. When glycan signature ions were detected in the HCD spectrum within 15 ppm mass accuracy, additional precursor isolation and MS/MS spectra were acquired using two methods:

1. The Ion Trap mass analyzer (*m/z* range: auto, scan rate: rapid) with 35% CID energy.
2. The Orbitrap mass analyzer (mass range: normal, scan range mode: define first mass *m/z* 120, resolution: 50,000 FWHM) with 25% EThcD collision energy.

### Data Analysis for Identification of Glycoproteins

Glycopeptide analysis was performed using Byonic v5.7.11 (Protein Metrics Inc., Cupertino, CA, USA) as previously described (23). Briefly, the acquired spectra were searched against a database of coding sequences identified in the genome of *H. exemplaris* (Proteome ID UP000192578 in UniProt). Searches were performed with the following parameters: the precursor mass tolerance was 10 ppm; the fragment mass tolerance was 20 ppm; trypsin as the digestion enzyme with a maximum of two missed cleavages; carbamidomethylation of cysteine as a fixed modification; oxidation of methionine as a variable modification; targeted FDR (strict) for PSMs was 1%. *N*-glycan modifications were assessed using the *N*-glycan 38 insect library supplemented with the following glycans: HexNAc (4)Hex(3)Fuc(3), HexNAc(3)Hex(3)Fuc(2), HexNAc(2)Hex(3)Fuc(1), HexNAc(5)Hex(3)Fuc(2), and HexNAc(4)Hex(3)Fuc(2). Glycopeptide spectral matches were stringently filtered, requiring a Byonic score ≥200 and |Log Prob| ≥ 2 to ensure high-confidence identifications. The output data from the Byonic search is presented in Supplemental Table S1, as it provides information on the glycopeptides and glycoproteins identified in the LC-MS/MS analysis.

### BLAST Analysis

Protein sequence similarity searches were conducted using the NCBI BLAST + tool (version 2.15.0), available at https://blast.ncbi.nlm.nih.gov/. The query protein sequences were aligned against the nr database (non-redundant protein sequences; downloaded January 2024). Searches were performed using BLASTp with the following parameters: an e-value threshold of 1e-5, a word size of 3, and default gap penalties. Taxonomic filters were applied to focus the search on *H. exemplaris*. The results were analyzed based on the Bit score, which normalizes the score of sequence alignments, to enable comparability across searches. Only alignments with a Bit score of ≥50 were considered, as this threshold effectively eliminated low-confidence matches.

### Experimental Design and Statistical Rationale

No biological and technical replicates were performed for the glycomics and glycoproteomics analyses due to the very limited amount of tardigrade samples available. However, the validity of the LC-MS analysis was confirmed by the consistent observation of major glycopeptides, even when analyzing samples at one-fifth of the original volume. This study focuses on a quantitative interpretation of glycoproteomics, demonstrating the presence of glycoproteins with the terminal Fucα1,3-GlcNAc motif.

## Results

### Identification of *N*-glycans Derived From Tardigrades

**Fig. 1:**
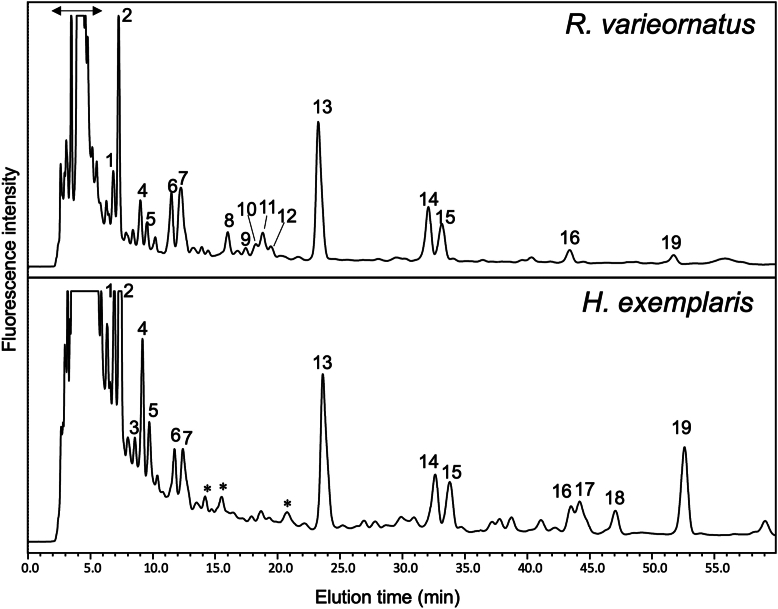
**HPLC *N*-glycan profiles on the ODS column derived from *R. varieornatus* and *H. exemplaris.*** The peaks were numbered in order of elution time. PA-oligosaccharides were assigned using Glycomod (https://web.expasy.org/glycomod/) with a mass tolerance of ±0.5 Da. Peaks unassignable to *N*-glycans are indicated by asterisks.

**Fig. 2:**
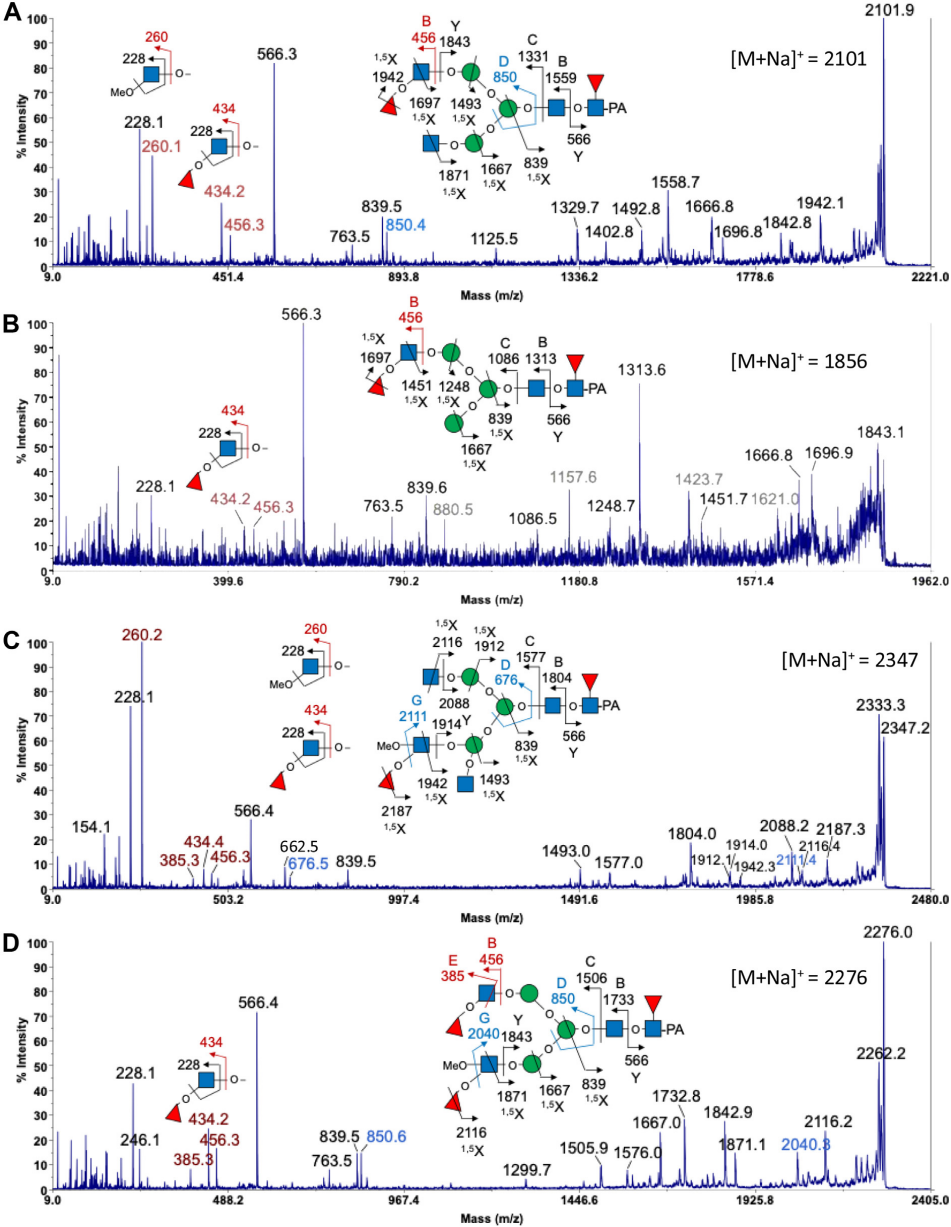
**MALDI MS/MS sequencing of the permethylated PA-glycans.** The permethylated PA-glycans found in fractions (*A*) 16, (*B*) 17, (*C*) 18, and (*D*) 19 were subjected to MS/MS fragmentation analysis. Each of the sodiated molecular ions afforded a series of sodiated reducing end ^1,5^X and Y ions, which collectively established their overall sequences. The non-reducing terminal units, either a HexNAc or a FucHexNAc, were identified by the oxonium ions at *m/z* 260 or 434 (annotated in *red*), respectively. The latter was additionally represented by sodiated B and E ions at *m/z* 456 and 385 (also annotated in *red*). The fragment ions unique to high energy CID cleavages on a MALDI TOF/TOF, including the E, G, and D ions, have been described previously (36) and were named accordingly. The D ions (annotated in blue) allow defining the 6-arm substituents on the trimannosyl core although alternative isomeric arrangements could not be ruled out. The cartoon drawings of the assigned glycan structures follow the recommended symbol nomenclature for glycans (37). The structures of the PA-glycans from fractions 16, 17, 18, and 19 were assigned based on MALDI MS/MS sequencing analysis as well as HPLC mapping combined with treatments using β-*N*-acetylhexosaminidase and/or α-fucosidase.

*N*-glycans from two species of tardigrades (*R. varieornatus* and *H. exemplaris*) in an anhydrobiotic state were released via hydrazinolysis, labeled with 2-aminopyridine, and subsequently subjected to multi-dimensional HPLC profiling. Figure 1 displays the *N*-glycosylation HPLC-profile on an octadecyl silica (ODS) column of the pyridylaminated (PA-) glycans. The PA-oligosaccharides were identified based on the coincidence of the elution data, presented as glucose units (GU) on columns calibrated with a PA-derivatized isomalto-oligosaccharides and mass values with those in the GALAXY database (Supplemental Fig. S1 and Table 2). However, PA-glycans corresponding to fractions 16, 17, 18, and 19 did not match any of the PA-glycans previously registered in GALAXY. However, PA-glycans containing di- or tri-deoxyHex, corresponding to fractions 16, 17, 18, and 19, did not match any previously registered PA-glycans in the GALAXY database. These PA-glycans were susceptible to α-fucosidase, and their identities were subsequently registered in GALAXY. For instance, the glycosyl composition of the major α-fucosylated PA-glycan in *H. exemplaris* found in fraction 19 could be assigned as (deoxyHex)_3_(Hex)_3_(HexNAc)_4_PA, based on its molecular mass of 1832 Da as determined by MALDI-TOF-MS. Upon α-fucosidase treatment, this glycan released three fucose residues, resulting in a biantennary oligosaccharide: GlcNAcβ1-2Manα1-6[GlcNAcβ1-2Manα1-3]Manβ1-4GlcNAcβ1-4GlcNAc-PA (code no. 200.1 in the GALAXY database) (Supplemental Fig. S2). Its precursor structure is, therefore, a 200.1 glycan extended with two non-reducing terminal Fuc residues and one core Fuc residue. In a similar manner, the positions of non-reducing terminal Fuc residue in the PA-glycans from fractions16, 17, and 18 were determined through HPLC mapping combined with treatments using β-*N*-acetylhexosaminidase and/or α-fucosidase (Table 2). To verify this assignment and further define the unusual fucosylation pattern, MALDI MS/MS sequencing was then performed on each of the permethylated derivatives of fractions 16, 17, 18, and 19 (Fig. 2).

**Table 1:** GC–MS linkage analysis of partially methylated alditol acetates prepared from the PA-glycans derived from *R. varieornatus* and *H. exemplaris*

*O*-methylated alditol acetates (glycosyl residue)^a^	Elution time (min)		Characteristic fragment ions (*m/z*)	Relative abundance^b^	
	*R. varieornatus*	*H.* e*xemplaris*		*R. varieornatus*	*H.exemplaris*
2,3,6-*O*-trimethyl-GlcNAc-ol (4-GlcNAc)	35.54	38.50	75, 99, 117, 143, 159, 233	1.00	1.00
2,4,6-*O*-trimethyl-GlcNAc-ol (3-GlcNAc)	40.49	40.44	75, 100,117, 129, 159, 161, 275	0.07	0.17
2,3,4,6-*O*-tetramethyl-GlcNAc-ol (Terminal-GlcNAc)	35.20	35.15	75, 117, 129, 145, 159, 161, 203, 205	0.88	0.88
2,3,4-O-trimethyl-Fuc-ol (Terminal-Fuc)	16.94	16.96	59, 102, 115, 118, 131, 175	0.06	0.10

a The glycosyl residues were identified based on the relative retention time and characteristic EI-MS fragment ions afforded by the respective partially methylated alditol acetates, with reference to authentic standards.

b The respective TIC peak areas were integrated for an approximate indication of their abundance relative to 4-GlcNAc, which was arbitrarily set as 1.00, without correcting for individual response factors.

**Table 2:** *N*-Glycans derived from *R. varieornatus* and *H*. *exemplaris*

Peak	GU (ODS)^a^	Observed mass value^b^	Structure	Relative quantity^c^ (mol %)	
				*R. varieornatus*	*H.exemplaris*
1	5.0	1821.6		4.2	5.5
2	5.2	1983.7		14.8	10.5
3	5.8	1659.7		-	1.2
4	6.1	1497.5		3.8	6.7
5	6.3	2145.6		1.8	3.2
6	7.1	1335.4		7.1	3.9
7	7.4	849.3		1.5	-
		1011.3		3.7	2.8
		1173.4		4.9	1.1
		1214.4		1.2	2.0
8^d^	8.4	979.4		2.4	-
9	8.8	1141.4		0.7	-
10	9.1	1417.5		1.2	-
11^d^	9.3	995.4		3.9	-
12	9.4	833.3		1.3	-
13	10.2	995.3		3.8	4.1
		1157.4		16.4	9.0
		1360.5		3.8	5.6
14	12.6	1563.5		9.7	6.5
15	12.9	1360.5		7.1	5.7
16	15.9	1709.9		2.4	3.1
17	16.2	1506.6		-	5.0
18	17.3	1912.8		-	3.2
19	19.3	1855.6		1.7	11.6
Others				2.6	9.3
Total				100	100

a GU were calculated from the elution times of the peaks obtained from the ODS column in Figure 1.

b Average mass observed as the m/z values of [M + Na]^+^ ions for PA-oligosaccharides by MALDI-TOF-MS. The molar percentage of individual glycans within a single peak was calculated based on the peak intensity in the MALDI-TOF-MS data.

c The ratio (mol %) was calculated from the peak area in Figure 1 by comparison with the total *N*-glycan content derived from *R.varieornatus* and *H. exemplaris*.

d The structure did not match any entry in the GALAXY database. Structural identification was achieved through a combination of MS/MS analysis and α-mannosidase digestion (Supplemental Figs. S4 and S5).

Among the most informative fragment ions commonly afforded by the sodiated molecular ions was the Y ion at *m/z* 566, which unambiguously identified the core fucosylation. It was complemented by the B ions at *m/z* 1559, 1313, 1804, and 1733, for the molecular ions detected in fractions 16, 17, 18, and 19, respectively, fully consistent with their deduced glycosyl compositions and unambiguously localized one Fuc to the reducing end PA-tagged GlcNAc. The non-reducing terminal Fuc-HexNAc moiety was, in turn, defined by the sodiated B ion at *m/z* 456. Notably, the corresponding Fuc-HexNAc ^+^ oxonium ion at *m/z* 434 was accompanied not by the loss of a MeOH moiety (−32 u) but by the elimination of a Fuc instead to yield the ion at *m/z* 228. This characteristic is strongly indicative of a Fuc 3-linked to GlcNAc (24). Further confirmation of the non-reducing terminal Fucα1,3-GlcNAc structures was provided by gas chromatography-mass spectrometry (GC-MS) linkage analysis of the total PA-glycan pool. In addition to the expected residues, including terminal Fuc (detected as 2,3,4-*O*-trimethyl-Fuc-ol) and 4-linked GlcNAc (2,3,6-*O*-trimethyl-GlcNAc-ol), 3-linked GlcNAc (2,4,6-*O*-trimethyl-GlcNAc-ol) and non-substituted terminal GlcNAc (2,3,4,6-*O*-tetramethyl-GlcNAc-ol) were also identified (Supplemental Fig. S3 and Table 1). The latter is consistent with structures deduced for fractions 16, 17, 18, and 19 (Table 2) in which one or both arms of the fucosylated trimannosyl core were extended by non-fucosylated terminal GlcNAc. In particular, MALDI MS/MS on fractions 16 and 18, but not fractions 17 and 19, afforded the HexNAc^+^ oxonium ion at *m/z* 260, whereas the FucHexNAc^+^ oxonium ion at *m/z* 456 was commonly detected in all four fractions analyzed (Fig. 2). It is evident that all four novel structures not previously registered in GALAXY carried the uncommon terminal Fucα3GlcNAc glycotope on either one or both arms of the trimannosyl core.

### Identification of Glycoproteins Possessing *N*-Glycans With Fucα1,3-GlcNAc Structure

**Fig. 3:**
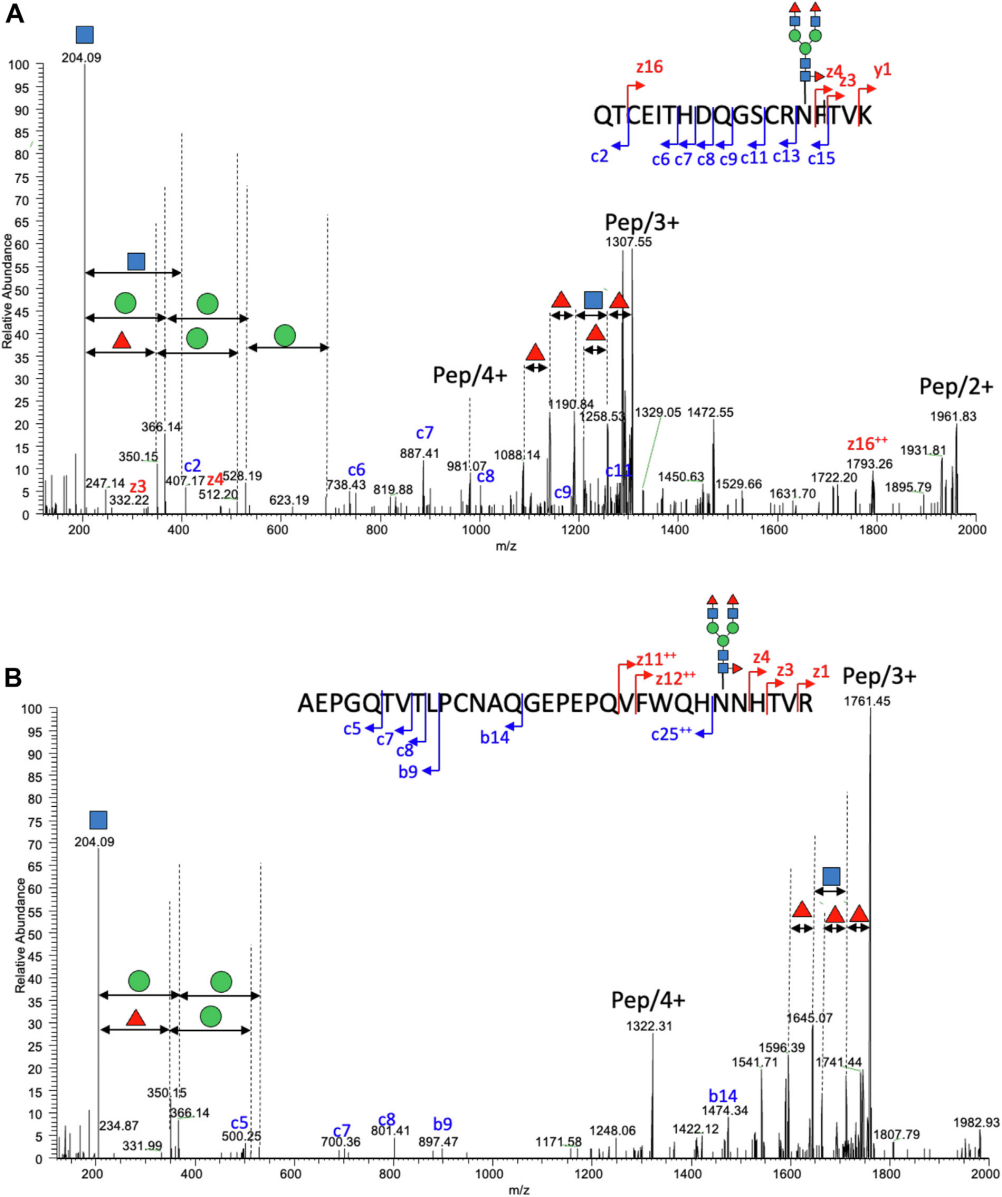
**MS/MS fragmentation of papilin *N*-glycopeptides containing the Fucα1-3GlcNAc structures.** Two examples of distinct glycopeptides are presented: (*A*) QTCEITHDQGSCRNFTVK and (*B*) AEPGQTVTLPCNAQGEPEPQVFWQHNNHTVR, both of which feature a HexNAc(4)Hex(3)Fuc(3) composition. EThcD fragmentation of these glycopeptides resulted in complete sequence coverage. The oxonium ion at m/z 350.15 [HexNAc(1)Fuc(1)] and fragment ions of the parent glycopeptide, excluding HexNAc(1)Fuc(2) and Fuc(2), confirm the presence of a glycan with the Fucα1,3-GlcNAc structure. According to the CFG nomenclature, *blue squares*, *green circles*, and *red triangles* indicate *N*-acetylglucosamine, mannose, and fucose, respectively.

To identify glycoproteins with the terminal Fucα1,3-GlcNAc motif, we conducted LC/MSMS analysis on protein fractions derived from *H. exemplaris* in an anhydrobiotic state. Proteins were extracted from approximately 10,000 tardigrades, then alkylated and digested with trypsin. The digested peptides were loaded onto an amide-80 column to collect the glycopeptide-enriched fraction. The glycoprotein fraction was subsequently subjected to LC/MS analysis. Figure 3 displays representative MS/MS spectra featuring an oxonium ion at *m/z* 350.15 corresponding to the [HexNAc(1)Fuc(1)] structure and fragment ions of the parent glycopeptide, excluding HexNAc(1)Fuc(2) and Fuc(2). This indicates the presence of a glycan with the Fucα1,3-GlcNAc antennae on glycopeptides 971 to 988 and 1579 to 1609 derived from papilin.

**Fig. 4:**
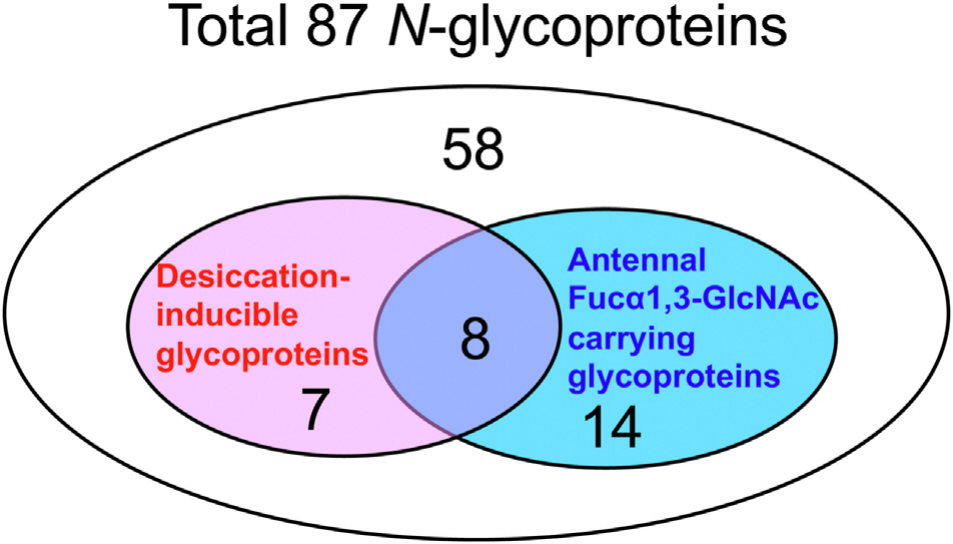
**AVenn diagram illustrates the identified*****N*****-glycoproteins.** The overlap between *N*-glycoproteins expressing the non-reducing terminal Fucα1,3-GlcNAc structure and those classified as proteins with increased expression during anhydrobiosis was illustrated.

To ensure the most confident glycopeptide identifications, we validated only PSMs (peptide-spectrum matches) that corresponded to electron-transfer/higher-energy collisional dissociation (EThcD) spectra and met strict thresholds: a non-negligible error probability |log Prob| >2.0 and a Byonic score >200. After applying stringent criteria, we identified a total of 8634 PSMs, leading to the discovery of 338 unique intact *N*-glycopeptides derived from 87 different glycoproteins in *H. exemplaris* (see Supplemental Table S2). Among these *N*-glycoproteins, 22 exhibited the Fucα1,3-GlcNAc antenna (refer to Fig. 4 and Table 3).

**Table 3:** List of *N*-glycoproteins identified in *H. exemplaris*

Non-desiccation-inducible glycoproteins	Gene name	Protein annotation	IUPred3 score^a^	Non-desiccation-inducible glycoproteins
Glycoproteins with Fucα1,3-GlcNAc antenna	BV898_01220	Superoxide dismutase (Cu-Zn)	0.25	Desiccation-inducible glycoproteins
	BV898_03883	hypothetical protein	0.74	
	BV898_00184	PPPDE domain-containing protein	0.37	
	BV898_10055	hypothetical protein	0.01	
	BV898_08163	Chitin-binding type-2 domain-containing protein	0.00	
	BV898_01100	WAP domain-containing protein	0.84	
	BV898_05466	Papilin	0.17	
	BV898_11928	hypothetical protein	0.70	
	BV898_01512	hypothetical protein	0.04	Non-desiccation-inducible glycoproteins
	BV898_01574	Transmembrane emp24 domain-containing protein 9	0.02	
	BV898_03535	FAD-linked oxidoreductase	0.01	
	BV898_04117	Vitellogenin-6	0.14	
	BV898_05246	GST N-terminal domain-containing protein	0.06	
	BV898_06105	G-protein coupled receptors family 2 profile 1 domain-containing protein	0.55	
	BV898_06922	CD109 antigen	0.11	
	BV898_06991	Neuroglian	0.32	
	BV898_08161	Chitin-binding type-2 domain-containing protein	0.00	
	BV898_08977	Pancreatic lipase-related protein 2	0.02	
	BV898_09795	L-sorbosone dehydrogenase	0.01	
	BV898_11190	Laminin subunit alpha	0.11	
	BV898_13158	Chitin-binding type-2 domain-containing protein	0.12	
	BV898_13788	Nidogen-1	0.17	
Glycoproteins without Fucα1,3-GlcNAc antenna	BV898_09026	Secreted protein	0.00	Desiccation-inducible glycoproteins
	BV898_12027	hypothetical protein	0.38	
	BV898_06777	von Willebrand factor A domain-containing protein	0.14	
	BV898_06906	MATH domain-containing protein	0.00	
	BV898_06776	von Willebrand factor A domain-containing protein	0.25	
	BV898_03546	hypothetical protein	0.10	
	BV898_00260	Sarcalumenin	0.26	
	BV898_00022	hypothetical protein	0.11	Non-desiccation-inducible glycoproteins
	BV898_00065	Endoplasmic reticulum-Golgi intermediate compartment protein 1	0.04	
	BV898_00095	hypothetical protein	0.50	
	BV898_00099	putative Apolipoprotein B-100	0.18	
	BV898_00100	Ectonucleoside triphosphate diphosphohydrolase 2	0.04	
	BV898_00577	putative Beta-1,3-galactosyltransferase 5	0.01	
	BV898_00612	hypothetical protein	0.18	
	BV898_00911	hypothetical protein	0.19	
	BV898_02207	putative Apolipoprotein D	0.01	
	BV898_02239	Dolichyl-diphosphooligosaccharide-protein glycosyltransferase subunit 1	0.63	
	BV898_02454	Mitochondrial ribonuclease P protein 3	0.03	
	BV898_02736	hypothetical protein	0.00	
	BV898_02837	Lysosomal alpha-glucosidase	0.02	
	BV898_02893	putative Pancreatic triacylglycerol lipase	0.00	
	BV898_03230	DBH-like monooxygenase protein 1-like protein	0.06	
	BV898_03257	putative Leucine-rich repeat-containing protein 15	0.00	
	BV898_03417	hypothetical protein	0.15	
	BV898_03425	Sodium/potassium-transporting ATPase subunit beta-2	0.07	
	BV898_03478	hypothetical protein	0.00	
	BV898_03587	hypothetical protein	0.08	
	BV898_03819	putative Ras association domain-containing protein 1	0.12	
	BV898_03853	hypothetical protein	0.01	
	BV898_04264	Cathepsin B	0.04	
	BV898_04276	Laminin subunit gamma-1	0.09	
	BV898_04951	Epoxide hydrolase 1	0.00	
	BV898_05042	hypothetical protein	0.09	
	BV898_05629	hypothetical protein	0.12	
	BV898_05653	hypothetical protein	0.08	
	BV898_06078	putative Zinc metalloproteinase nas-13	0.12	
	BV898_06174	Serpin B9	0.00	
	BV898_06377	hypothetical protein	0.09	
	BV898_06476	putative Testicular acid phosphatase-like protein	0.18	
	BV898_06985	hypothetical protein	0.69	
	BV898_07240	hypothetical protein	0.03	
	BV898_07373	putative CD63 antigen	0.00	
	BV898_07785	hypothetical protein	0.04	
	BV898_08327	putative Insulin-like growth factor-binding protein complex acid labile subunit	0.00	
	BV898_08330	Vitellogenin-6	0.09	
	BV898_08568	Alkyldihydroxyacetonephosphate synthase, peroxisomal	0.05	
	BV898_08580	Arylsulfatase J	0.17	
	BV898_08836	putative Translocon-associated protein subunit alpha	0.14	
	BV898_09465	putative Translocon-associated protein subunit beta	0.06	
	BV898_09643	hypothetical protein	0.00	
	BV898_09705	Cullin-4A	0.03	
	BV898_10022	hypothetical protein	0.08	
	BV898_10209	hypothetical protein	0.13	
	BV898_10240	hypothetical protein	0.24	
	BV898_10963	hypothetical protein	0.21	
	BV898_11681	Protein mesh	0.15	
	BV898_11843	Dynein heavy chain 7, axonemal	0.04	
	BV898_12405	putative Carbonic anhydrase 14	0.14	
	BV898_12554	Integrin alpha-PS2	0.09	
	BV898_12667	Chitinase-3-like protein 1	0.00	
	BV898_12870	hypothetical protein	0.19	
	BV898_13159	hypothetical protein	0.00	
	BV898_13336	hypothetical protein	0.22	
	BV898_13836	hypothetical protein	0.46	
	BV898_14266	Multidrug resistance-associated protein 1	0.10	

a IUPred3 score of 0.5 or more are shown in red. IUPred3 is a tool that identifies intrinsically disordered protein regions using a biophysics-based model, providing a score between 0 and one for each residue to indicate the likelihood of that residue being part of a disordered region (35).

Based on the previous study that analyzed RNA sequencing data from fully hydrated and post-desiccation samples from *H. exemplaris* (25), a total of 15 glycoproteins showing increased expression levels were identified and presented in Table 3. Among these, eight contained the Fucα1,3-GlcNAc antenna (Fig. 4 and Table 3).

## Discussion

The *N*-glycosylation profiles of the two tardigrade species, *R. varieornatus* and *H. exemplaris*, consist of high-mannose-type, paucimannose-type, and complex-type oligosaccharides with no detectable hybrid-type glycans. In both species, paucimannose-type oligosaccharides are among the notable components, accounting for 39% in *R. varieornatus* and 17% in *H. exemplaris.* Paucimannose-type oligosaccharides are widely found in insects and nematodes, which also belong to Ecdysozoa (26). In the tardigrade species, a significant portion of the paucimannose-type oligosaccharides undergoes fucosylation of the innermost GlcNAc residue, exclusively via α1,6-linkage. This type of core fucosylation is commonly observed in animals (27). Additionally, insects and nematodes are known to possess difucosyl core structures, where two fucose residues are attached to the 3- and 6-positions of the innermost GlcNAc (28). However, the results of HPLC and LC-MS analyses (Fig. 1 and Supplemental Fig. S6) indicate that such glycans are rarely expressed in *H*. *exemplari*, and represent only a minor component in *R*. *varieornatu*. Moreover, charged glycans were not detected as major components in our HPLC analysis. Consequently, these minor glycans were excluded from the focus of this study.

In contrast, we identified uncommon complex-type glycans in the tardigrade species. Specifically, the complex-type oligosaccharides identified in this study lack galactosylation but undergo α1,3-fucosylation at the non-reducing terminal GlcNAc residues.

Antenna fucosylation with an α1,3-linkage is common in mammals, exemplified by the Lewis X antigen, but is less prevalent in invertebrates and varies significantly among species. In mammals, FUT9 is responsible for Lewis X formation, while in *Schistosoma mansoni*, this role is fulfilled by FUT-F. Both enzymes require Galβ1,4-GlcNAc as an acceptor substrate (29). FucTC from the honeybee *Apis mellifera* and the mosquito *Anopheles gambia* catalyze α1,3-fucosylation of GalNAcβ1,4-GlcNAc as acceptor, producing an antennal Lewis-like GalNAcβ1,4(Fucα1,3)GlcNAc moiety (30). This underscores the distinctive nature of the non-reducing terminal Fucα1,3-GlcNAc structure observed in tardigrades, which lacks both Galβ1,4 and GalNAcβ1,4 modifications.

The antennal Fucα1,3-GlcNAc structures, devoid of Gal or GalNAc, have been identified on *N*-glycans in *Taenia crassicephalum* and *Manduca sexta* (31, 32). Additionally, a recent MS analysis detected similar *N*-glycans with a fucosyl GlcNAc antenna as minor glycans in some nematodes (33, 34). These findings suggest the existence of an α1,3-fucosyltransferase with unique substrate specificity in these organisms. A key finding of this study is that the *N*-glycans with this distinctive structure are prevalent in *H. exemplaris*.

This distinctive glycosylation is more prevalent in *H. exemplaris*. Among the 87 *N*-glycoproteins identified from *H. exemplaris*, 22 glycoproteins expressed the Fucα1,3-GlcNAc structure (Fig. 4). Notably, eight of these glycoproteins were induced during anhydrobiosis, including Cu/Zn-superoxide dismutase (SOD) and papilin (Table 3).

The SOD enzyme is an antioxidant that is secreted extracellularly and located between tardigrade cells, protecting the organism from oxidative stress. Conversely, papilin is thought to promote the reconstruction and remodeling of extracellular matrix by regulating the activity of metalloproteases. SOD has a single *N*-linked glycosylation site, to which a biantennary complex-type glycan with or without the non-reducing terminal Fucα1,3-GlcNAc structure was attached. For paplin, out of the 21 potential *N*-linked glycosylation sites, glycan information was obtained for two sites. All the detected paplin glycopeptides possessed *N*-glycans containing the Fucα1,3-GlcNAc antenna. The functions and localization of these protective proteins may be influenced by their *N*-glycosylation patterns.

Among the eight glycoproteins with desiccation-inducible properties that contain the Fucα1,3-GlcNAc antenna, three—annotated as BV898_03883, BV898_01100, and BV898_11928—exhibited high IUPred3 scores. This tool identifies intrinsically disordered protein regions (IDRs) using a biophysics-based model (35). Notably, BV898_01100 and BV898_11928 have been confirmed to display *N*-glycans featuring the Fucα1,3-GlcNAc antenna at regions predicted to be IDRs. In tardigrades, several proteins with IDRs are supposed to play roles in the cryptobiosis mechanism. Consequently, these glycoproteins may also be critical during desiccation, potentially leveraging their distinctive *N*-glycans to recruit proteins with lectin activity, among other functions, thereby significantly contributing to this process.

Tardigrade genomic analyses identified several genes encoding homologs of FUT9 (Uniport entry name: FUT9_HUMAN) and FucTC (Uniport entry name: Q05GU1_APICA) (Supplemental Tables S3 and S4). Recombinant expression of these candidate enzymes, along with recently developed techniques for gene expression and suppression in tardigrades, will enable elucidation of the molecular mechanisms underlying their distinctive glycosylation. While our study did not involve glycoproteomic analysis of active tardigrades, and therefore whether the Fucα1,3-GlcNAc-containing glycans themselves are upregulated during desiccation remains to be elucidated, this work provides a crucial foundation for future investigations into the role of glycans in tardigrade anhydrobiosis.

## Data Availability

The data presented in this study are available upon request from the corresponding authors. The LC data, along with the subsequent MALDI-TOF-MS data, and the MS-based glycoproteomics data have been deposited in GlycoPOST (Accession No. GPST000534) and JPOST (Accession No. JPST003355), respectively.

## Supplemental Data

This article contains Supplemental Data.

## Conflicts of Interests

The authors declare that they have no conflicts of interest with the contents of this article.

## Supporting Information

Supplemental Table S1

Supplementarl Figs. S1–S6 and Tables S4 and S5
