# Supplementary material for: Uncommon N-Glycan Structures in Anhydrobiotic Tardigrades
Source: Mol Cell Proteomics. 2025 Apr 28;24(6):100979. doi: 10.1016/j.mcpro.2025.100979 (PMC12167791; doi:10.1016/j.mcpro.2025.100979)
Supplement: Supplementarl Figs. S1–S6 and Tables S4 and S5 [file mmc2.docx]

**Supplemental Fig. S1**


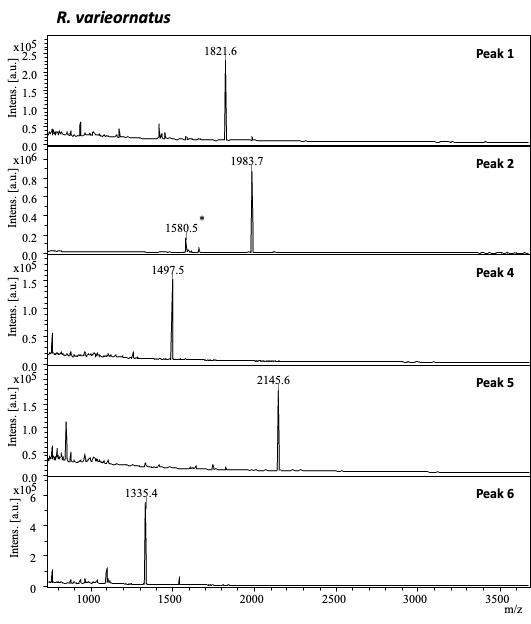


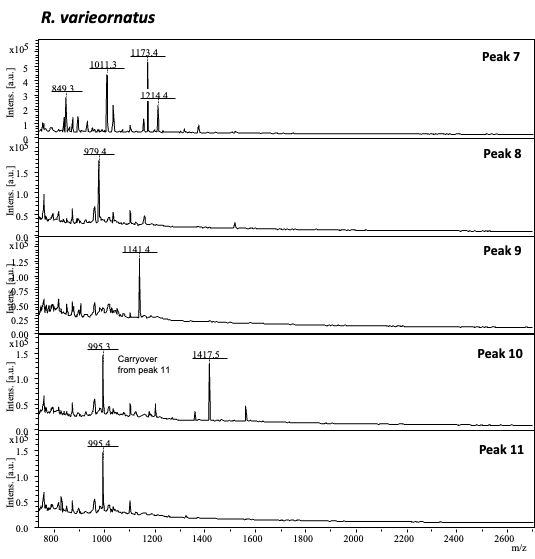


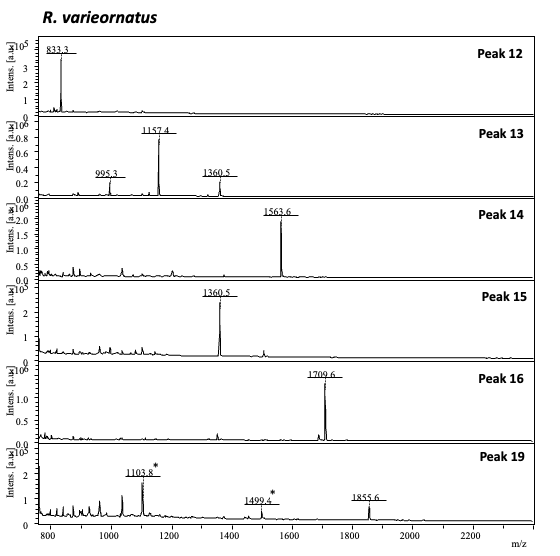


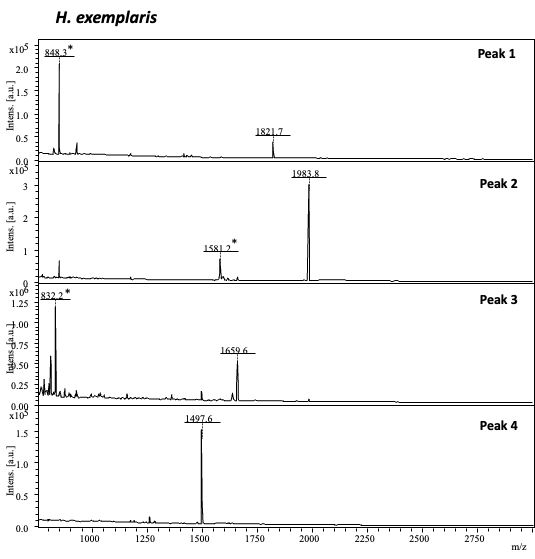


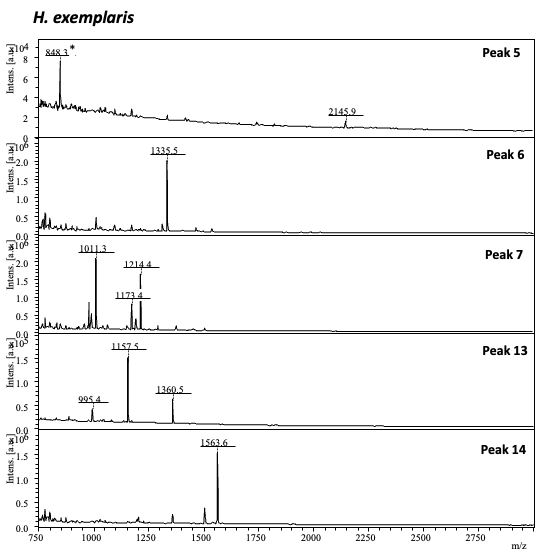


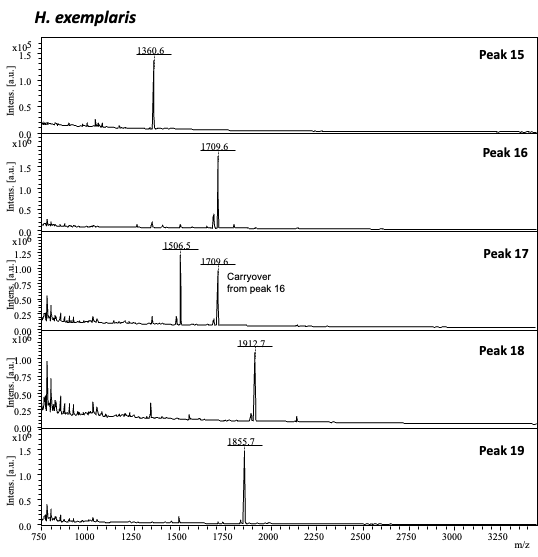


**Supplemental Fig. S1**: MALDI-TOF-MS spectra of *N*-glycans derived from tardigrades *R. varieornatus* and *H. exemplaris*. Glycan assignments for the detected peaks were performed using Glycomod (https://web.expasy.org/glycomod/) with a mass tolerance of ±0.5 Da. Asterisked peaks, which could not be assigned to *N*-glycans within this mass tolerance, were excluded from further consideration as *N*-glycans and were categorized as unrelated components.

**Supplemental Fig. S2**

**Supplemental Fig. S2:** HPLC profiles of the *N*-glycan in 19 fraction assigned as (deoxyHex)_3_(Hex)_3_(HexNAc)_4_PA following α-fucosidase treatment, both with and without co-injection of standard *N*-glycans. The magenta trace illustrates the HPLC profile of the sample treated with α-fucosidase, while the blue trace represents the co-injection with standard *N*-glycans (code nos. 200.1 and 210.1 in the GALAXY database). Due to the low glycan content following fucosidase treatment, the reaction mixture was injected directly without re-purification. In the CFG nomenclature, blue squares, red triangle, and green circles denote *N*-acetylglucosamine, fucose and mannose, respectively.

**Supplemental Fig. S3**

**Supplemental Fig. S3**: GC/MS analysis of partially per-*O*-methylated, per-*O*-acetylated alditol acetate derivatives of PA-glycans derived from *H. exemplaris*. (Upper) Gas chromatograms illustrate the extracted ion chromatogram of representative fragment ions of 2,4,6-*O*-methyl-GlcNAc-ol. (Lower) MS fragmentation profiles of peaks eluting at 11.3 min corresponding to 2,4,6-*O*-methyl-GlcNAc-ol.

**Supplemental Fig. S4**

**
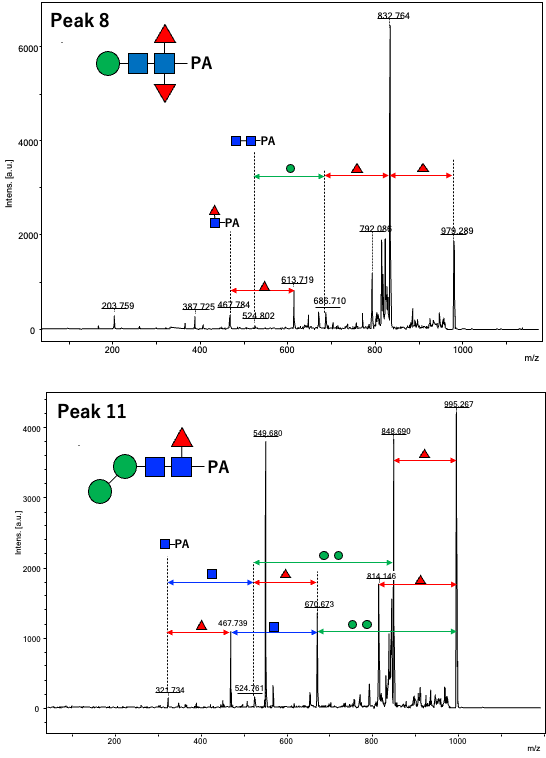
**

**Supplemental Fig. S4:** MS/MS spectra of *N*-glycans from the peak 8 and peak 11 fractions. In the CFG nomenclature, blue squares, red triangles, and green circles denote *N*-acetylglucosamine, fucose and mannose, respectively.

**Supplemental Fig. S5**

**
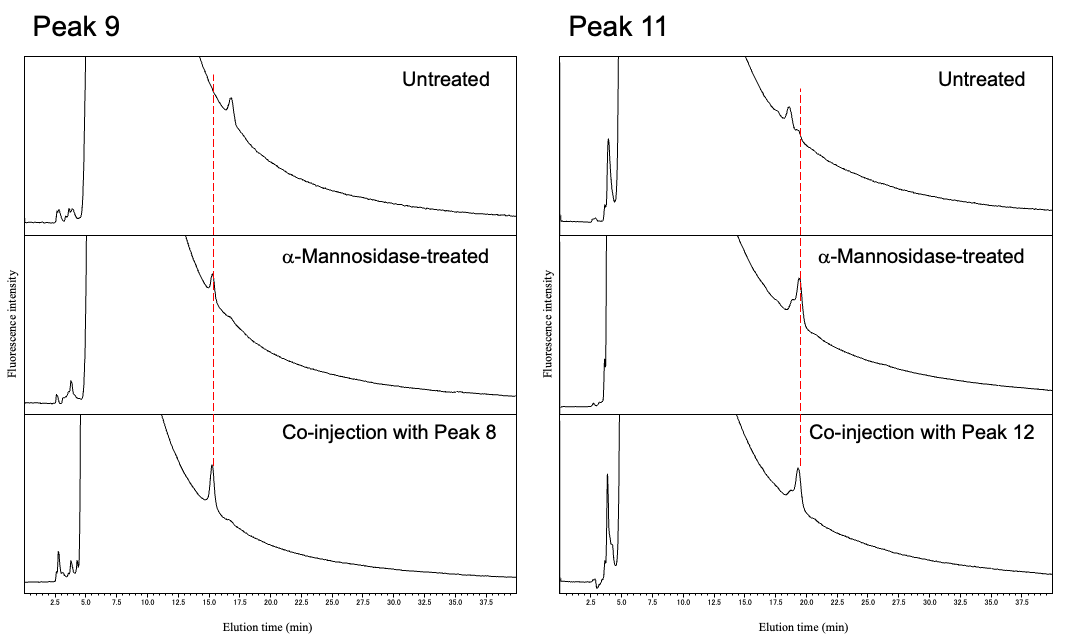
**

**Supplemental Fig. S5: Structural confirmation of *N*-glycans using α-mannosidase treatment.**

The digestion of the *N*-glycans from the peak 9 and peak 11 fractions with α-mannosidase confirmed that their resulting structures matched those of peaks 8 and 12, respectively. The identities of these two glycans were further validated by co-injection. Due to the low glycan yield after α-mannosidase treatment, the reaction mixture was analyzed without additional purification.

**Supplemental Fig. S6**

**Supplemental Fig. S6:**  Extracted ion chromatograms (EICs) of glycopeptides derived from *H. exemplaris*. The chromatograms display four distinct m/z ranges corresponding to diagnostic ions: (A) 204.0857–204.0877 (HexNAc(1)), (B) 350.1427–350.1463 (HexNAc(1)Fuc(1)), (C) 496.1925–496.1975 (HexNAc(1)Fuc(2), core difucosylation: Fuc(α1-3)Fuc(α1-6)GlcNAc), and (D) 512.1948–512.2000 (Hex(1)HexNAc(1)Fuc(1), terminal Fucα1,3-GlcNAc). LC-MS analysis of the diagnostic ion associated with di-Fuc cores (496 m/z) revealed minimal peak detection. Furthermore, the MS/MS spectra containing the 496 m/z ion did not show the corresponding diagnostic ion for glycopeptides (204 m/z). These findings suggest that glycans with difucosyl cores, if present in tardigrades, are likely to exist as minor structural components.

| **Supplemental Table S3**: Genes encoding homologs of *FUT9* (Uniport entry name: FUT9_HUMAN) in *H. exemplaris.* | | |
| --- | --- | --- |
|  |  |  |
| Gene_ID | Entry | Bit score |
| BV898_19383 | A0A9X6NLM2 | 129 |
| BV898_00666 | A0A1W0XE39 | 125 |
| BV898_00665 | A0A1W0XE57 | 123 |
| BV898_11186 | A0A1W0WHJ8 | 121 |
| BV898_11602 | A0A1W0WGB8 | 119 |
| BV898_14855 | A0A9X6NJ46 | 118 |
| BV898_06970 | A0A1W0WUM0 | 117 |
| BV898_15651 | A0A9X6RKQ0 | 114 |
| BV898_11601 | A0A1W0WGC8 | 112 |
| BV898_01728 | A0A1W0XAX1 | 112 |
| BV898_01632 | A0A1W0XB79 | 110 |
| BV898_14769 | A0A9X6NGI8 | 110 |
| BV898_07229 | A0A1W0WUB7 | 108 |
| BV898_07882 | A0A1W0WSD5 | 108 |
| BV898_08283 | A0A1W0WR43 | 106 |
| BV898_04616 | A0A1W0X1M7 | 97.4 |
| BV898_17103 | A0A9X6RMH9 | 97.1 |
| BV898_00664 | A0A1W0XE40 | 82 |
| BV898_01156 | A0A1W0XBS4 | 65.5 |
| BV898_05591 | A0A1W0WYL1 | 63.5 |
| BV898_19848 | A0A9X6RQ46 | 61.2 |
| BV898_16439 | A0A9X6RLM0 | 58.2 |
| BV898_00663 | A0A1W0XE36 | 56.6 |
| BV898_19999 | A0A9X6RQ00 | 49.7 |
| BV898_16438 | A0A9X6RLU0 | 40.4 |

| **Supplementaｌ Table S4**: Genes encoding homologs of *FucTC* (Uniport entry name: Q05GU1_APICA) in *H. exemplaris.* | | |
| --- | --- | --- |
|  |  |  |
| Gene_ID | Entry | Bit score |
| BV898_01728 | A0A1W0XAX1 | 215 |
| BV898_08283 | A0A1W0WR43 | 197 |
| BV898_14855 | A0A9X6NJ46 | 194 |
| BV898_06970 | A0A1W0WUM0 | 192 |
| BV898_11186 | A0A1W0WHJ8 | 189 |
| BV898_15651 | A0A9X6RKQ0 | 181 |
| BV898_11602 | A0A1W0WGB8 | 180 |
| BV898_07229 | A0A1W0WUB7 | 177 |
| BV898_00665 | A0A1W0XE57 | 176 |
| BV898_11601 | A0A1W0WGC8 | 166 |
| BV898_19383 | A0A9X6NLM2 | 166 |
| BV898_01632 | A0A1W0XB79 | 163 |
| BV898_17103 | A0A9X6RMH9 | 163 |
| BV898_00666 | A0A1W0XE39 | 162 |
| BV898_04616 | A0A1W0X1M7 | 146 |
| BV898_07882 | A0A1W0WSD5 | 119 |
| BV898_05591 | A0A1W0WYL1 | 113 |
| BV898_16439 | A0A9X6RLM0 | 105 |
| BV898_14769 | A0A9X6NGI8 | 103 |
| BV898_00664 | A0A1W0XE40 | 102 |
| BV898_01156 | A0A1W0XBS4 | 93.6 |
| BV898_00663 | A0A1W0XE36 | 79 |
| BV898_16438 | A0A9X6RLU0 | 50.4 |
